# Statement of Retraction: Dexmedetomidine attenuates ischemia and reperfusion-induced cardiomyocyte injury through p53 and forkhead box O3a (FOXO3a)/p53-upregulated modulator of apoptosis (PUMA) signaling signaling

**DOI:** 10.1080/21655979.2024.2299625

**Published:** 2024-02-20

We, the Editors and Publisher of the journal *Bioengineered*, have retracted the following article:

Feng Yun Yang, Lu Zhang, Yan Zheng & He Dong (2022) Dexmedetomidine attenuates ischemia and reperfusion-induced cardiomyocyte injury through p53 and forkhead box O3a (FOXO3a)/p53-upregulated modulator of apoptosis (PUMA) signaling signaling, Bioengineered, 13:1, 1377–1387, DOI: 10.1080/21655979.2021.2017611

Since publication, significant concerns have been raised about the integrity of the data and reported results in the article. When approached for an explanation, the authors did not provide their original data or any necessary supporting information. As verifying the validity of published work is core to the integrity of the scholarly record, we are therefore retracting the article. All authors listed in this publication have been informed. The authors have agreed to retract the article. Moreover, the authors have subseqeuntly informed us that they identified a further issue and requested retraction.

We have been informed in our decision-making by our editorial policies and the COPE guidelines.

The retracted article will remain online to maintain the scholarly record, but it will be digitally watermarked on each page as ‘Retracted’.

